# Evaluation eines strukturierten E‑Learning-basierten Ansatzes zur Vermittlung der CT-Anatomie der Nasennebenhöhlen bei Medizinstudierenden: Eine Pilotstudie

**DOI:** 10.1007/s00106-021-01141-x

**Published:** 2022-01-18

**Authors:** Anna Marleen Krahe, Manuel Christoph Ketterer, Christian Offergeld, Tanja Hildenbrand

**Affiliations:** Klinik für Hals‑, Nasen- und Ohrenheilkunde, Universitätsklinikum Freiburg, Killianstr. 5, 79106 Freiburg, Deutschland

**Keywords:** Radiologische Anatomie, Präoperative CT-Beurteilung, CLOSE-Kriterien, Anatomische Normvarianten, Endoskopische Nasennebenhöhlenchirurgie, Radiologic anatomy, Preoperative CT evaluation, CLOSE criteria, Anatomical variants, Endoscopic sinus surgery

## Abstract

**Hintergrund:**

Die radiologische Anatomie ist in vielen Ländern kein integraler Bestandteil des Medizinstudiums. Die Radiologie scheint für neue online-basierte Lehrmethoden besonders geeignet.

**Ziel der Arbeit:**

Das Ziel dieser Studie ist es, die Eignung des E‑Learnings zur Einführung komplexer Lerninhalte, wie der CT-Anatomie (radiologische Anatomie in der Computertomographie), zu prüfen und zu prüfen, ob die Identifikation wichtiger anatomischer Normvarianten durch Medizinstudent(inn)en durch eine Computertomographie-Checkliste verbessert werden kann.

**Material und Methoden:**

Medizinstudierende wurden gebeten, Computertomographien (CT) der Nasennebenhöhlen auf anatomische Normvarianten vor und nach der Einführung der CLOSE-Kriterien (cribriform plate = Lamina cribrosa, Lamina papyracea, Onodi-Zelle = Sphenoethmoidale Zelle, sphenoid sinus = Keilbeinhöhle, ethmoidal artery = Arteria ethmoidalis anterior) zu untersuchen. Sowohl die CT-Anatomie als auch die CLOSE-Kriterien wurden mittels E‑Learning vermittelt. Die Rate der korrekt identifizierten Normvarianten und die Ergebnisse für die einzelnen CLOSE-Items wurden ermittelt. Die subjektive Evaluation des Nutzens der Checkliste und des E‑Learnings erfolgte mithilfe eines Fragebogens.

**Ergebnisse:**

Zehn Studierende nahmen an dieser Pilotstudie teil. Die Rate der korrekt identifizierten anatomischen Normvarianten verbesserte sich nach der Einführung der CLOSE-Kriterien signifikant von 33,3 auf 61,1 %. Die Analyse der einzelnen CLOSE-Items zeigte eine signifikante Verbesserung für C, S und E. Die subjektive Evaluation des E‑Learnings und der CT-Checkliste waren sehr positiv.

**Schlussfolgerung:**

Komplexe Lerninhalte können Medizinstudierenden mittels E‑Learning vermittelt werden, auch wenn bisher kein Vorwissen in diesem Bereich besteht. Das E‑Learning wird als angemessene Methode zur Einführung der Thematik beurteilt. Ein strukturiertes Vorgehen mithilfe der CLOSE-Kriterien kann die Erkennung anatomischer Normvarianten signifikant verbessern.

## Abstract (en)

**Background:**

Computed tomography (CT) anatomy is not an integral part of undergraduate medical training in many countries. Radiology seems to be well suited for new online-based teaching methods.

**Objective:**

The aim of this study was to evaluate whether e‑learning is appropriate for introducing complex learning contents such as sinus CT anatomy to novices and to assess whether identification of relevant anatomical variants in sinus CT scans by medical students can be improved with a sinus CT checklist.

**Materials and methods:**

Medical students were asked to assess sinus CT scans for anatomical variants before and after implementation of the CLOSE mnemonic (cribriform plate, lamina papyracea, Onodi cell, sphenoid sinus pneumatization, and [anterior] ethmoidal artery). Sinus CT anatomy and the CLOSE mnemonic were introduced by e‑learning. The rate of correctly identified variants and the results of the individual CLOSE items were recorded. A questionnaire was distributed for subjective evaluation of the usefulness of the checklist and e‑learning.

**Results:**

Ten students took part in this pilot study. The rate of correctly identified variants improved significantly, from 33.3 to 61.1%. The analysis of the individual CLOSE items showed a significant improvement for C, S, and E. The subjective evaluation of the CLOSE mnemonic and e‑learning was very positive.

**Conclusion:**

E‑learning was able to transfer complex learning contents in previously non-trained medical students and was evaluated as an appropriate introduction to the topics. Structured assessment of paranasal sinus CT scans using the CLOSE criteria can significantly improve the recognition of anatomical variants.

Die chronische Rhinosinusitis ist eine der häufigsten Erkrankungen der Nasennebenhöhlen und die einzige HNO-Erkrankung, die als wichtig und repräsentativ genug erachtet wurde, um in den nationalen kompetenzbasierten Lernzielkatalog für das Medizinstudium als sog. Fokuserkrankung aufgenommen zu werden. Zur Diagnose und Beurteilung des Ausmaßes der Erkrankung, zur Operationsplanung und Verringerung des Risikos für intraoperative Komplikationen ist die Kenntnis der CT-Anatomie der Nasennebenhöhlen unerlässlich. Im Medizinstudium wird der radiologischen Anatomie nur wenig Raum eingeräumt.

## Hintergrund

Die Lehre der radiologischen Anatomie im Allgemeinen und der radiologischen Anatomie in der Computertomographie (CT-Anatomie) der Nasennebenhöhlen im Besonderen ist in vielen Ländern unzureichend [4, 8, 16]. Die Einführung in die Interpretation von Computertomographien (CT) erfolgt in der Regel im Rahmen der klinischen Tätigkeit von Medizinstudierenden und der Weiterbildungszeit. Die Deutsche Röntgengesellschaft hat auf nationaler Ebene ein Curriculum für die radiologische Lehre an deutschen medizinischen Fakultäten vorgeschlagen [3]. Für den Bereich der Nase und Nasennebenhöhlen beinhaltet dieses die wichtigsten anatomischen Strukturen und das Basiswissen über die häufigsten radiologischen Befunde traumatischer und entzündlicher Erkrankungen. Die European Society of Radiology hat wesentliche Inhalte für das Medizinstudium erarbeitet, um Standards in der radiologischen Lehre in Europa zu verbessern und zu vereinheitlichen. Bei Erkrankungen aus dem Hals‑, Nasen- und Ohrenbereich (HNO) sollten Erkrankungen der Nasennebenhöhlen abgedeckt werden [4]. Die European Society of Radiology hat zudem ein Statement zu neuen Lehrmethoden in der radiologischen Lehre herausgegeben [5]. Dieses empfiehlt die Integration von Methoden wie E‑Learning und problembasiertem Lernen zur Vermittlung radiologischer Lehrinhalte im Medizinstudium.

Die Radiologie scheint für neue Online-Lehrmethoden besonders geeignet. In Zeiten der Corona-Pandemie mit geschlossenen Universitäten und abgesagten Präsenzveranstaltungen sind Online-Lehrformate unverzichtbar. E‑Learning ist in der medizinischen Lehre weit verbreitet. Es erlaubt eine maximale Unabhängigkeit durch die flexible Organisation von Zeit und Raum. Die Lerninhalte können so oft wie nötig wiederholt werden. Studien konnten zeigen, dass E‑Learning genauso effektiv sein kann wie traditionelle Lehrmethoden und dass die radiologische Anatomie und Kenntnisse der Bildinterpretation durch E‑Learning erfolgreich vermittelt werden können [1, 6, 7, 18, 29].

Checklisten sind inzwischen auch in der Medizin weitverbreitet. Sie werden in allen medizinischen Bereichen eingesetzt und durch die Weltgesundheitsorganisation (WHO) unterstützt. Sie stellen einen effektiven und systematischen Ansatz zur Verbesserung der Patient(inn)ensicherheit in der Chirurgie dar [12, 27]. Checklisten werden zudem erfolgreich in der medizinischen Ausbildung eingesetzt, vor allem im Rahmen von leistungsbasierten Prüfungen, wie z. B. der objektiven strukturierten klinischen Untersuchung und der OSCE (Objective Structured Clinical Examination) [11, 15].

Es existieren Checklisten, die speziell für die Nasennebenhöhlenchirurgie entwickelt wurden [22–25].

Die CLOSE-Kriterien zur Evaluation von CT-Bildern der Nasennebenhöhlen wurden erstmals von Weitzel et al. beschrieben [28]. Sie sind kurz und leicht zu reproduzieren. Es konnte gezeigt werden, dass die Identifikation anatomischer Strukturen und Normvarianten in CT-Bildern durch die strukturierte Beurteilung mittels dieser Kriterien verbessert werden kann [2, 9, 30].

- „*C*ribriform plate“: Keros-Klassifikation, Asymmetrie, knöcherne Dehiszenz der Schädelbasis
- *L*amina papyracea: Dehiszenz, Prolaps von Orbitainhalt, infraorbitale Zelle, Processus uncinatus mit Kontakt zur Lamina papyracea
- *O*nodi-Zelle (sphenoethmoidale Zelle): vorhanden/nicht vorhanden, Verlauf des N. opticus in Onodi-Zelle
- „*S*phenoid sinus“ (Keilbeinhöhle): Pneumatisation, Dehiszenz des Karotis- und/oder Optikuskanals, Keilbeinhöhlenseptum mit Ansatz am Karotiskanal
- „*E*thmoidal artery“ (Arteria ethmoidalis anterior): Identifikation des Eintritts in das Siebbein (zipflige Ausziehung an der medialen Wand der Orbita), Verlauf durch das Siebbein (in der Schädelbasis, frei durch das Siebbein verlaufend)

Das Ziel dieser Studie ist es, folgende Hypothesen zu prüfen:

- Durch die Anwendung einer CT-Checkliste mit Abarbeitung der CLOSE-Kriterien erhöht sich die Rate der korrekt identifizierten anatomischen Normvarianten in der CT der Nasennebenhöhlen signifikant.
- Das E‑Learning führt zu einer adäquaten Vermittlung der Grundlagen der CT-Beurteilung bei Medizinstudierenden ohne Vorerfahrung.
- Der Einsatz der CLOSE-Kriterien und das E‑Learning werden von Studierenden positiv bewertet.

## Material und Methoden

### E-Learning

**Figure 1:**
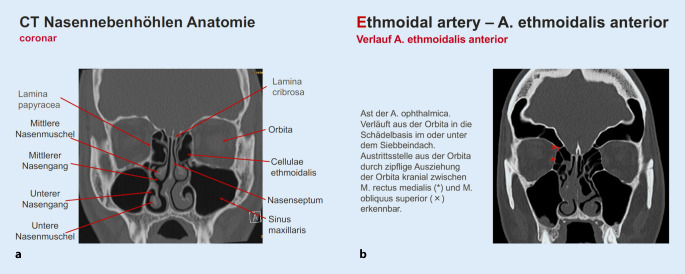

Das zweiteilige E‑Learning wurde speziell für diese Studie erstellt. Der erste Teil enthielt eine Wiederholung der Anatomie der Nasennebenhöhlen, Grundlagen der Bildgebung, die CT-Anatomie der Nasennebenhöhlen und CT-Befunde anatomischer Normvarianten sowie eine Erläuterung ihrer klinischen Relevanz. Das E‑Learning enthielt Folien mit theoretischem Inhalt und CT-Beispielen (Abb. 1a,b).

Der zweite Teil des E‑Learnings diente der Einführung der CLOSE-Kriterien. Die unterschiedlichen Strukturen wurden auf CT-Beispielbildern erläutert und deren klinische Relevanz ausgeführt.

### Studienteilnehmer(innen)

Medizinstudierende im 5. Studienjahr wurden gebeten, an der Studie teilzunehmen. Es handelte sich um ein extracurriculares Angebot, an dem die Studierenden freiwillig teilnehmen konnten. Es wurden erfahrene Studierende gewählt, da sie bereits über eine ausreichende Kenntnis der Anatomie und ein fortgeschrittenes Niveau theoretischer medizinischer Kenntnisse verfügen, das mit dem junger Ärzt(inn)e(n) vergleichbar ist, bei jedoch noch geringer klinischer und insbesondere chirurgischer Erfahrung. Sie sind deshalb gut geeignet, um die Effektivität des E‑Learnings in der Vermittlung komplexer Lerninhalte, wie der CT-Anatomie der Nasennebenhöhlen zu untersuchen. Zuvor wurden lediglich Basis-Kenntnisse der CT-Anatomie im Rahmen der Vorlesung Nase/Nasennebenhöhlen, einer gemeinsamen Vorlesung der Anatomie, Radiologie und HNO vermittelt.

### Studienablauf

Nach der Einwilligung in die Studienteilnahme wurde der erste Teil des E‑Learnings per E‑Mail verschickt. Während einer ersten Sitzung wurden jedem Studierenden 10 anonymisierte CT der Nasennebenhöhlen von Patient(inn)en in 2 bis 3 Ebenen (axial, koronar, wenn vorhanden sagittal) vorgelegt. Diese sollten auf anatomische Normvarianten geprüft werden. Eine Beurteilung der entzündlichen Veränderungen sollte nicht erfolgen. Die CT-Bilder wurden in einem regulären klinischen Bildbetrachtungsprogramm (IMPAX EE R20 VIII, Fa. Agfa HealthCare N. V, Mortsel, Belgien) präsentiert, in dem die Studierenden in den verschiedenen Ebenen durch die Bilder scrollen konnten. Insgesamt waren in den Bildern 18 anatomische Normvarianten vorhanden. Jedem Studierenden stand ein eigener Computer zur Verfügung, und es bestand keine zeitliche Begrenzung für die Beurteilung der Bilder. Die Nutzung der Unterlagen des E‑Learnings war nicht gestattet. Die Studierenden wurden gebeten, die identifizierten anatomischen Normvarianten für jede CT frei zu benennen. Dies wurde durch einen unabhängigen Untersucher dokumentiert, ohne dass die richtige Lösung preisgegeben wurde.

Nach dieser Sitzung wurde das zweite E‑Learning-Modul wieder per E‑Mail bereitgestellt. In der zweiten Sitzung wurden die Studierenden gebeten, jeweils die identischen 10 CT mithilfe der CLOSE-Kriterien als CT-Checkliste (ohne dass diese direkt vorlag) erneut zu beurteilen. Um das Risiko eines möglichen Lerneffekts bei der wiederholten Beurteilung der gleichen CT-Bilder zu reduzieren, wurde den Studierenden nach der ersten Sitzung nicht erläutert, ob ihre Antworten richtig waren, und es lagen mindestens 4 Wochen zwischen beiden Sitzungen. Die CT waren passwortgeschützt gespeichert und für die Studierenden nicht zugänglich, um die Möglichkeit des Austauschs zwischen den Studierenden zu minimieren. Die Studierenden wurden erneut gebeten, die anatomischen Normvarianten in jeder CT zu benennen, und dies wurde von einem unabhängigen Untersucher dokumentiert.

Nach der zweiten Sitzung wurde den Studierenden ein subjektiver Evaluationsbogen zum Nutzen der Checkliste und des E‑Learnings (modifiziert nach [30]) vorgelegt. Die Elemente des Fragebogens sind in Tab. 1 dargestellt. Diese wurden auf einer Likert-Skala bewertet (1 = stimme ich völlig zu, 2 = stimme ich eingeschränkt zu, 3 = stimme ich nicht zu, 4 = ich weiß nicht).

**Table 1:** 

Fragen	Beurteilung
Frage 1	Die präoperative Checkliste ist sinnvoll
Frage 2	Die Checkliste stellt sicher, dass ich mir genug Zeit für die Betrachtung der CT-Bilder nehme
Frage 3	Die Checkliste macht mich sicherer im Umgang mit der Anatomie und der Computertomographie der Nasennebenhöhlen
Frage 4	Das E‑Learning war angemessen für die Einführung in die Themen

*CT* Computertomographie

### Ethische Gesichtspunkte

Die Studie wurde im Einklang mit nationalem Recht und der Deklaration von Helsinki in ihrer aktuellen Fassung durchgeführt. Die Arbeit wurde durch die Ethikkommission der Albert-Ludwigs-Universität Freiburg genehmigt (Ethikkommission Freiburg; Nummer: 204/19).

Den Teilnehmenden wurde ein Informationsblatt zur Studie zur Verfügung gestellt und sie gaben ihre schriftliche Einwilligung zur Teilnahme an der Studie. Sie wurden zusätzlich über ihre Rechte gemäß der aktuellen Datenschutzgrundverordnung informiert und gaben ihr schriftliches Einverständnis zur Sammlung, Analyse und Speicherung personenbezogener Daten.

### Statistische Analyse

Die Anzahl der korrekt identifizierten anatomischen Normvarianten (in %) vor der Einführung der CLOSE-Kriterien wurde mit dem Wert nach der Einführung verglichen. Achtzehn Normvarianten wurden als 100 % gewertet. Zudem wurden die Normvarianten den einzelnen CLOSE-Kriterien zugeordnet und separat ausgewertet. In den 10 CT konnten 2 Varianten der Lamina cribrosa, 6 der Lamina papyracea, 2 der Keilbeinhöhle und 2 der A. ethmoidalis anterior zugeordnet werden. Es waren zudem 4 CT-Bilder mit Onodi-Zellen enthalten. Zwei CTs enthielten eine Concha bullosa media, die zur Gesamtzahl der anatomischen Normvarianten gezählt, jedoch nicht den verschiedenen CLOSE-Kriterien zugerechnet wurde, da sie hierin nicht aufgeführt ist.

Die statistische Analyse erfolgte mit IBM SPSS Statistics (Fa. IBM Corp. 2015. IBM SPSS Statistics für Windows, Version 24.0, Armonk, NY, USA).

Die Daten wurden mittels vorgeschalteten Levene-Tests untersucht, um eine homo- versus heterogene Varianz zu definieren. Außerdem wurde ein t‑Test für verbundene Stichproben berechnet zur Ermittlung von statistisch signifikanten Unterschieden. Der *p*-Wert für statistische Signifikanz wurde auf < 0,05 festgelegt. Die Grafiken wurden mit Excel (Microsoft, Redmond, WA, USA) erstellt.

Die Ergebnisse des Evaluationsbogens wurden deskriptiv ausgewertet.

Minimum, Maximum, Mittelwert und Standardabweichung wurden für jede Frage berechnet.

## Ergebnisse

Zehn Studierende (4 männlich, 6 weiblich) wurden in die Studie eingeschlossen und nahmen an beiden Testsitzungen teil. Das durchschnittliche Alter der Studierenden betrug 25,4 Jahre. Jeder Studierende untersuchte 10 CT vor und nach der Einführung der CLOSE-Kriterien und füllte den Evaluationsbogen aus.

**Figure 2:**
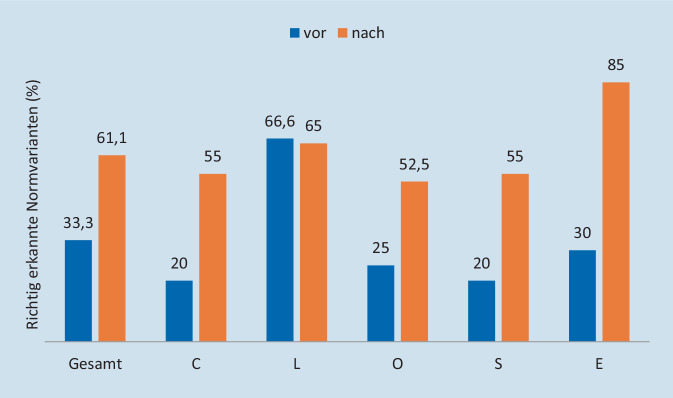

Insgesamt wurden während der ersten Sitzung 33,3 % der anatomischen Normvarianten von der Gesamtgruppe richtig erkannt (Min. 5,6 %, Max. 55,6 %). Nach der Einführung der CLOSE-Kriterien wurden 61,1 % der anatomischen Normvarianten von der Gesamtgruppe der Studierenden korrekt identifiziert (Min. 38,9 %, Max. 88,9 %) (Abb. 2). Nach der Einführung der CLOSE-Kriterien verbesserte sich die Erkennung anatomischer Normvarianten signifikant (*p* < 0,0001).

Die Ergebnisse der einzelnen CLOSE-Kriterien sind in Abb. 2 dargestellt. Es zeigte sich eine signifikante Verbesserung für *C* (Lamina cribrosa), *S* (Keilbeinhöhle) und *E* (A. ethmoidalis anterior). Die größte prozentuale Verbesserung zeigte sich für anatomische Varianten der A. ethmoidalis anterior.

Die Ergebnisse der subjektiven Evaluation sind in Tab. 2 zusammengefasst. Alle Studierende fanden die CLOSE-Kriterien sinnvoll, und 70 % gaben an, dass sie sicherstellen, dass sie sich genug Zeit für die Beurteilung der CT-Bilder nehmen. 90 % der Studierenden stimmten vollkommen zu, und 10 % stimmten eingeschränkt zu, dass sie die Kriterien sicherer im Umgang mit der Anatomie und CT-Bildern der Nasennebenhöhlen machen. Die Hälfte der Studierenden stimmte vollkommen zu, und die andere Hälfte stimmte eingeschränkt zu, dass das E‑Learning eine angemessene Einführung in das Thema darstellt. Die Tab. 2 zeigt die Ergebnisse der Evaluation für alle Einzelfragen mit Mittelwerten und Standardabweichung.

**Table 2:** 

–	Minimum (Anzahl Studierende)	Maximum (Anzahl Studierende)	Mittelwert	SD
Frage 1	1 (10)	1 (10)	1	± 0
Frage 2	1 (7)	4 (1)	1,5	± 0,972
Frage 3	1 (9)	2 (1)	1,1	± 0,316
Frage 4	1 (5)	2 (5)	1,5	± 0,527

*SD* Standardabweichung

1 = Stimme ich vollkommen zu, 2 = Stimme ich eingeschränkt zu, 3 = Stimme ich überhaupt nicht zu, 4 = Ich weiß nicht

## Diskussion

Die CT-Anatomie ist kein integraler Bestandteil des Medizinstudiums in Deutschland und vielen anderen Ländern [8, 16]. Die meisten Studierenden besitzen am Ende ihres Studiums nur wenig Vorwissen und Erfahrung in der Interpretation von CT-Bildern und insbesondere der CT-Anatomie der Nasennebenhöhlen. Die Kenntnis der CT-Anatomie ist Voraussetzung für die Erkennung und Interpretation krankhafter Veränderungen.

Verschiedene Studien beurteilen die Eignung des E‑Learnings für die Vermittlung radiologischer Kenntnisse. Die meisten untersuchen die Interpretation von einfachen Röntgenbildern, z. B. des Thorax. Sie konnten zeigen, dass die Interpretationsfähigkeiten verbessert werden können und dass die Studierenden das E‑Learning für ein adäquates Medium hielten, um den Lerninhalt zu vermitteln [18, 21, 29]. Nur wenige Studien zeigen positive Resultate für komplexere radiologische Bildgebungstechniken wie Notfall-CT des Gehirns und PET-CT der Lunge bei Tumoren [6, 7]. Die vorliegende Studie beurteilt die Interpretation von CT-Bildern der Nasennebenhöhlen. Diese ist komplexer als die Beurteilung einfacher zweidimensionaler Röntgenbilder, da durch das Durchscrollen der Bilder in verschiedenen Ebenen eine dritte Dimension hinzugefügt wird. Zusätzlich untersuchen die meisten verfügbaren Studien die Fähigkeit, pathologische Befunde zu erkennen. Diese Studie untersucht die Erkennung anatomischer Normvarianten. Sie sind weniger offensichtlich als pathologische Befunde und so schwieriger zu identifizieren.

In der ersten Sitzung erkannten die Studierenden 33,3 % der anatomischen Normvarianten in den vorgelegten CT-Bildern. Diese Rate ist bereits höher als bei Assistenzärzt(inn)e(n) in einer Studie von Error et al. und einer vorangegangenen Studie unserer Arbeitsgruppe [2, 9]. Unter Kenntnis der curricularen Lehrinhalte sind die Vorkenntnisse der Studierenden dieser Studie als gering einzustufen. Für die Beurteilung einiger Normvarianten ist die Interpretation mehrerer Ebenen notwendig. Die Studierenden konnten die Lerninhalte aus zweidimensionalen Beispielbildern des E‑Learnings auf bewegte Bilder in verschiedenen Ebenen in der CT übertragen. Die Ergebnisse deuten darauf hin, dass das E‑Learning geeignet scheint, auch komplexe Inhalte wie die CT-Anatomie vermitteln zu können.

Das Fehlen von IT-Kenntnissen wurde kontrovers als Hindernis für den Erfolg des E‑Learnings in der Lehre im Gesundheitswesen bzw. der HNO identifiziert [17, 20, 26]. Die meisten Studierenden in westlichen Ländern sind jedoch mit verschiedenen IT-Anwendungen vertraut und können als ausreichend ausgerüstet für neue Online-Lehrmethoden angesehen werden. Die Studierenden dieser Studie gaben keine Schwierigkeiten mit der Bearbeitung der Lehrinhalte an.

Ein strukturiertes Vorgehen bei der CT-Beurteilung durch die Nutzung der CLOSE-Kriterien als CT-Checkliste kann die Identifikation kritischer anatomischer Normvarianten signifikant verbessern. Dies konnte bereits bei Assistenzärzt(inn)en verschiedener Ausbildungsstadien gezeigt werden [9]. Error et al. konnten ebenfalls eine statistisch signifikante Verbesserung der Identifikation wichtiger anatomischer Strukturen in CT der Nasennebenhöhlen nach Einführung der CLOSE-Kriterien bei 9 Jung- und 9 Altassistent(inn)en nachweisen [2]. In einer einfach verblindeten Studie wurden die Assistenzärzt(inn)e(n) durch einen erfahrenen Facharzt zu Beginn einer Operation befragt und aufgefordert, kritische anatomische Strukturen der Patient(inn)en vor und nach der Einführung der CLOSE-Kriterien zu benennen. Sie analysierten 57 präoperative Gespräche (28 vor und 29 nach der Implementierung der CLOSE-Kriterien). Mit einer Gesamtzahl von 100 CT-Beurteilungen jeweils vor und nach der Einführung der Checkliste prüft die vorliegende Studie eine deutlich größere Anzahl. Assistenzärzt(inn)e(n) verfügen im Gegensatz zu Studierenden, abhängig vom Ausbildungsstand, über ein gewisses Vorwissen und Erfahrung in der Beurteilung von CT-Bildern. Nach der Einführung der CLOSE-Kriterien verbesserte sich die Erkennung anatomischer Normvarianten signifikant von 33,3 auf 61,1 %. Das strukturierte Vorgehen anhand der CLOSE-Checkliste verbesserte somit auch bei Studierenden mit geringen Vorkenntnissen die Identifikation anatomischer Normvarianten.

Es gibt nur wenige Studien, die den Nutzen von Checklisten in der medizinischen Ausbildung bewerten. Eine Studie von Hofer et al. konnte zeigen, dass die Einführung einer Checkliste im Rahmen des Präparierkurses das Lernergebnis und die Dissektionsqualität verbesserten [10]. Andere Studien untersuchten ihren Nutzen bei leistungsbasierten Prüfungen, wie der OSCE. Diese ist validiert und in der medizinischen Lehre etabliert. Checklisten werden im Rahmen der OSCE zur strukturierten und objektiven Beurteilung der praktischen Leistung der Studierenden genutzt [15]. Im Gegensatz dazu wurden die CLOSE-Kriterien in dieser Studie als Checkliste genutzt, um medizinisches Wissen zu vermitteln und anzuwenden. Das strukturierte Vorgehen mittels Checklisten wurde in einer ausreichenden Zahl von Studien und Probanden zur OSCE validiert [11]. Die vorliegende Studie konzentriert sich zunächst auf 10 Studierende, um zu zeigen, dass ein strukturiertes Vorgehen mithilfe der Checkliste grundsätzlich geeignet scheint, die Vermittlung von Wissen bei ungeschulten Proband(inn)en zu verbessern.

Error et al. analysierten zusätzlich die einzelnen CLOSE-Kriterien und fanden eine signifikante Verbesserung für alle Punkte außer der Lamina papyracea [2]. Dies zeigte sich auch in der aktuellen Studie. Mit 66,6 % korrekt identifizierter Normvarianten der Lamina papyracea zeigten die Studierenden bereits bei der ersten Sitzung gute Ergebnisse. Die Ergebnisse vor der Einführung der CLOSE-Kriterien sind mit den Ergebnissen von Error et al. vergleichbar, nach der Einführung im Vergleich jedoch schlechter. Dies könnte durch die Tatsache erklärt werden, dass es sich in der Studie von Error et al. um Assistenzärzt(inn)e(n) mit Erfahrung in der Interpretation von CT-Bildern handelte, da sie sich möglicherweise in einer Rotation mit dem Schwerpunkt Nase/Nasennebenhöhlen befanden. Hierdurch hätten sie alltäglichen Kontakt zu CT-Bildern der Nasennebenhöhlen. Diese Vorerfahrung ist bei den Studierenden der vorliegenden Studie nicht vorhanden. Die Ergebnisse deuten darauf hin, dass die Erkennung kritischer anatomischer Strukturen durch eine strukturierte Beurteilung mithilfe einer Checkliste verbessert werden kann, dass jedoch auch der wiederkehrende Kontakt mit CT der Nasennebenhöhlen notwendig zu sein scheint. Die zudem auch in dieser Studie erkennbare positive Beeinflussung durch Repetition und Anzahl von Trainingsmaßnahmen korreliert mit den Ergebnissen von Polk et al. bezüglich Präsenzveranstaltungen als auch mit der Studie von Krauss et al. für virtuelle Lehrformate [13, 19].

Eine subjektive Evaluation des Nutzens der CLOSE-Kriterien und des E‑Learnings wurde in die Studie integriert. Die Anwendung der Checkliste wurde von den Studierenden positiv beurteilt. Eine Studie von Yao et al. untersuchte den pädagogischen Wert und die Effektivität einer präoperativen CT-Checkliste der Nasennebenhöhlen aus der Perspektive von Assistenzärzt(inn)en [30]. Ihre Resultate sind mit denen dieser Studie vergleichbar. Die meisten Assistenzärzt(inn)e(n) in der Studie von Yao et al. waren der Meinung, dass die Checkliste nützlich sei, sie sicherer im Umgang mit der CT-Anatomie der Nasennebenhöhlen mache und helfe, kritische anatomische Strukturen zu identifizieren. In einer vorangegangenen Studie gaben Assistenzärzt(inn)e(n) der hiesigen HNO-Klinik ein ähnliches Feedback zur CLOSE-Checkliste [9].

Die Studierenden empfanden das E‑Learning als eine angemessene Einführung in die Themen. Studierende in anderen Studien zum E‑Learning zur Interpretation von radiologischen Bildern zeigen ebenfalls positive subjektive Evaluationen [21, 29].

Eine Limitation dieser Studie liegt in dem Einschluss einer kleinen Zahl von Studierenden nur einer medizinischen Fakultät. Die Studierenden haben sich möglicherweise zur freiwilligen Teilnahme an der Studie bereiterklärt, da sie ein spezielles Interesse an der HNO und extracurricularen Aktivitäten besitzen. Dies könnte einen Selektionsbias darstellen. Da die grundsätzliche Eignung des E‑Learnings geprüft werden sollte, wurde auf eine Kontrollgruppe verzichtet. Um diesen genannten Limitationen zu entgegnen, soll in einem nächsten Schritt die Vermittlung radiologischer Kenntnisse mittels E‑Learning mit traditionellen Lehrmethoden randomisiert in einer größeren Gruppe von Studierenden eines gesamten Semesters verglichen werden. Weitere Studien sollten zudem prüfen, ob sich die Ergebnisse in Studierendenpopulationen anderer Universitäten reproduzieren lassen.

Ein Lerneffekt zwischen der ersten und zweiten Sitzung ist nicht vollständig auszuschließen. Durch das Design der Studie wurde versucht, dieses Risiko zu minimieren. Die CT-Bilder waren zwischen den Sitzungen nicht zugänglich. Nach der ersten Sitzung wurden die richtigen Lösungen nicht bekannt gegeben. Eine Verbesserung der Ergebnisse durch Selbststudium bzw. mehrfaches Wiederhohlen des E‑Learnings kann durch das Studiendesign nicht ausgeschlossen werden. Andererseits ist die zeitlich und örtlich ungebundene Möglichkeit der Wiederholung auch ein Vorteil der Wissensvermittlung durch E‑Learning. Da eine signifikante Verbesserung nachgewiesen werden konnte, sind wir zuversichtlich, dass die Verbesserung nicht nur durch die Wiederholung, sondern v. a. durch das strukturierte Vorgehen mithilfe der CLOSE-Checkliste bedingt ist.

Der Einsatz der entwickelten E‑Learning-Module könnte auch in der Ausbildung von Weiterbildungsassistent(inn)en sinnvoll sein, da sie ein zeit- und ortsunabhängiges Lernen ermöglichen. Dies ist bei der begrenzten Zeit während der Weiterbildung von großer Bedeutung. Wie eine Studie von Linke et al. zeigen konnte, sind Weiterbildungsassistent(inn)en bereit, sich aktiv an der Ausbildung zu beteiligen, beurteilen die zur Fortbildung zur Verfügung stehende Zeit jedoch als kritisch [14].

## Fazit für die Praxis

- Komplexe Lerninhalte wie die CT(Computertomographie)-Anatomie können mittels E‑Learning an Studierende ohne wesentliches Vorwissen und mit wenig klinischer Erfahrung erfolgreich vermittelt werden.
- Die Identifikation anatomischer Normvarianten in der CT der Nasennebenhöhlen kann durch ein strukturiertes Vorgehen mittels einer CT-Checkliste zur Beurteilung wichtiger anatomischer Strukturen weiter verbessert werden.
- Die Vermittlung des Lehrinhaltes mittels E‑Learning und die Nutzung der CLOSE-Checkliste werden von den Studierenden positiv bewertet.

## Abkürzungen

CT: Computertomographie
CT-Anatomie: radiologische Anatomie in der Computertomographie
HNO: Hals‑, Nasen- und Ohrenheilkunde
OSCE: Objective Structured Clinical Examination
SD: Standardabweichung
WHO: World Health Organization
